# Medically unexplained pain complaints are associated with underlying unrecognized mood disorders in primary care

**DOI:** 10.1186/1471-2296-11-17

**Published:** 2010-03-03

**Authors:** Luis Agüera, Inmaculada Failde, Jorge A Cervilla, Paula Díaz-Fernández, Juan Antonio Mico

**Affiliations:** 1 Psychiatry Department, University Hospital 12 de Octubre, Complutense University, Madrid, Spain; 2 Preventive Medicine and Public Health Department, University of Cádiz, Spain; 3 Department of Psychiatry, University of Granada, Centro de Investigación Biomédica en Red de Salud Mental, CIBERSAM, Granada, Spain; 4 Medical Department, Boehringer-Ingelheim Spain; 5 Department of Neuroscience, Pharmacology and Psychiatry, School of Medicine, University of Cádiz, Centro de Investigación Biomédica en Red de Salud Mental, CIBERSAM, Cádiz, Spain

## Abstract

**Background:**

Patients with chronic pain frequently display comorbid depression, but the impact of this concurrence is often underestimated and mistreated. The aim of this study was to determine the prevalence of unrecognized major depression and other mood disorders and comorbid unexplained chronic pain in primary care settings and to explore the associated factors.

Also, to compare the use of health services by patients with unexplained chronic pain, both with and without mood disorder comorbidity.

**Methods:**

A cross-sectional study was carried out in a sample of primary care centers. 3189 patients consulting for "unexplained chronic pain" were assessed by the Visual Analogue Scales (VAS) and the Primary Care Evaluation of Mental Disorders (PRIME-MD) questionnaire.

**Results:**

We report: a) a high prevalence of unrecognized mood disorders in patients suffering from unexplained chronic pain complaints (80.4%: CI 95%: 79.0%; 81.8%); b) a greater susceptibility of women to mood disorders (OR adjusted = 1.48; CI 95%:1.22; 1.81); c) a direct relationship between the prevalence of mood disorders and the duration of pain (OR adjusted = 1.01; CI 95%: 1.01; 1.02) d) a higher comorbidity with depression if the pain etiology was unknown (OR adjusted = 1.74; CI 95%: 1.45; 2.10) and, e) an increased use of health care services in patients with such a comorbidity (p < 0.0001).

**Conclusions:**

The prevalence of undiagnosed mood disorders in patients with unexplained chronic pain in primary care is very high, leading to dissatisfaction with treatment processes and poorer outcomes. Consequently, it seems necessary to explore this condition more regularly in general practice in order to reach accurate diagnoses and to select the appropriate treatment.

## Background

Chronic pain and depression are amongst the most common health problems reported by patients attending primary care [1]. Both conditions produce increased use of health resources and impaired health-related quality of life [2,3].

Chronic pain and depression are frequently comorbid processes, with some studies showing a prevalence ranging from 15% to 100% [4]. Likewise, the clinical presence and severity of chronic pain are recognized as predictors of the degree of depression in these patients [5,6] with the association of both processes producing a greater impact on the patient than either disorder alone [5,7].

Patients with comorbid chronic pain and depression consult their doctor more often and become heavy consumers of medical services [8,9]. Furthermore, some studies suggest that patients with depression and chronic pain are more likely to be dissatisfied with their healthcare[10].

In primary care, depression is often undiagnosed, particularly in cases of patients suffering from chronic pain [11]. The WHO study on psychological disorders in general health care found that primary care physicians detected only 39.1% of cases of current depression [12]. Bair et al [4] demonstrated that physicians in these settings fail to accurately diagnose at least 50% of patients with major depression and that the treatment of somatic complaints often takes precedence over the identification and treatment of depression [13].

From a neurobiological perspective, chronic pain and depression share similar neurobiological mechanisms and central nervous structures. For example, serotonin and noradrenaline are implicated in both processes, and it is well known that antidepressants that enhance serotonin and noradrenaline are effective analgesics [14-16]. More recently, neuroimaging studies have supported this relationship [17,18].

In spite of the overwhelming data demonstrating the relationship between chronic pain and depression, studies concerning the comorbidity of chronic pain, especially unexplained chronic pain, with other mood disorders are scarce. Most studies have focused on pain's relationship with Major Depressive Disorder (MDD), with few addressing other mood conditions such as minor depression, dysthymia or bipolar disorder included in the *Diagnostic and Statistical Manual of Mental Disorders Fourth Edition *(DSM-IV). Likewise, studies using specific structured interviews to assess these processes in primary care are not common.

By means of a structured interview, this study investigated the prevalence of unrecognized mood disorders, not only MDD, in patients with medically unexplained chronic pain complaints in primary care settings, and also studied possible factors associated with the comorbid mood disorder. As a secondary outcome we aimed to compare the use of healthcare services in unexplained chronic pain patients with and without comorbid mood disorder.

## Method

### Study Design and Sampling Process

A cross-sectional study was carried out in a sample of primary care centers in Spain between April 2006 and December 2006. In order to obtain a representative sample, the number of primary care centers chosen in each Spanish region was proportionate to its number of inhabitants. Likewise, the selection process observed Spain's rural/urban ratio with at least 20% of the sample from primary care centers in cities of less than 50,000 inhabitants.

In each primary care center, one general practitioner (GP) that voluntarily accepted to participate was selected, constituting a final sample of 600 GPs.

The study was conducted in agreement with the Helsinki Declaration and with standard working procedures and protocols, and approved by the Clinical Research Ethics Committee at the Clinical and Provincial Hospital in Barcelona, ensuring adherence to the norms of good clinical practice.

### Patient Population

The study included men and women over 18 years of age who consulted their Primary Care Center for unexplained pain (head, neck, back, limbs or joints) lasting for at least 6 weeks. Thus, patients were considered with "unexplained chronic pain" and eligible for the study if the cause of their pain was either unknown or, in known, the cause was not fully explained by another medical or psychiatric disorder [19]. The presence of pain for 6 weeks or more was considered to be the criterium for chronic pain for the purpose of this study. Likewise, patients did not have a current diagnosis of any mood disorder in their medical record; they had to be mentally and physically able to participate in the study and had to give their written, informed consent.

### Sample Size

To obtain a prevalence of unexplained chronic pain and concomitant mood disorders of 20% with an accuracy of 3% and a confidence interval of 95%, based on the literature [20], the minimum number of patients was 683. A sample size of 3285 patients was calculated to be able to detect differences between sub-groups defined by age, sex and treatment type considering an OR = 1.5 with a confidence level of 95%, a power of 80%. This number assumed a probability of exposure to the factor under study of 15% in the group with concomitant mood disorder and also a ratio of 4 patients with mood disorders versus 1 without. Assuming a possible 10% drop-out rate due to incomplete data and absentees the final size necessary for the study was established at 3641 patients.

### Recruitment

To obtain the calculated number of patients each of the 600 selected GPs had to interview about 7 patients who consulted them and who fulfilled the inclusion criteria for the study. The patients were selected consecutively based on arrival at the primary care center, and patients who refused to participate were substituted for the next one who fulfilled the criteria.

### Measurements

The interviews were performed at local primary care facilities by GPs who received a written guideline with instructions on the procedure to be used to collect that data. Information related to socio-demographic variables (age, sex, and educational level), clinical variables (duration of pain, location of pain, pain treatment i.e. analgesics and/or psychotropics, and quality and duration of sleep) and use of healthcare resources (number of visits to doctors and hospital as a consequence of the pain in the previous 6 weeks/diagnostic tests related to the pain in the previous 6 weeks) was collected from a structured questionnaire and from the medical records of the patients.

The intensity of pain was measured with a Visual Analogical Scale (VAS) with a range of 0-100, where 0 was no pain and 100 was the worst pain possible [21]. For the detection and assessment of mood disorders the module of mood state from the Primary Care Evaluation of Mental Disorders (PRIME-MD) questionnaire was used. This questionnaire, designed by Spitzer et al [22] to help in the diagnosis of the most commonly observed mood disorders observed specifically in primary care, is based on the diagnostic criteria of the *Diagnostic and Statistical Manual of Mental Disorders Fourth Edition *(DSM-IV) and their sensibility and specificity are 83% and 88% respectively. A Spanish validated version of the PRIME-MD was used [23].

All possible PRIME-MD diagnoses according DSM-IV criteria were considered in our study: major depressive disorder, minor depressive disorder, partial remission from a major depressive disorder, dysthymic disorder, bipolar disorder or depression caused by a general medical condition, medication or drugs.

Data collection forms were further monitored centrally to check and correct missing data or inconsistencies.

### Statistical Analysis

In the descriptive analysis, absolute frequency and 95% CIs were calculated for categorical variables, and central trend and dispersion measurements were used for quantitative variables. The T- test, Wilcoxon test or chi-squared test (Student or Fisher) were used to study associations.

The prevalence (CI 95%) of undiagnosed mood disorder was calculated as the number of patients with an undiagnosed illness detected with the PRIME-MD questionnaire divided by the total number of patients screened with the PRIME-MD questionnaire.

To study the factors associated with mood disorders, a backward logistic regression model was used in which the outcome variable was the presence or absence of mood disorders, and the variables included in the model were: sex, age, location of pain (head, neck, back, joints), intensity of pain, duration of pain expressed as the percentage of the day in pain, number of pain locations, known cause of the pain (yes/no) and current treatment (non-steroidal anti-inflammatory drugs (NSAIDs), analgesic opioids, anticonvulsants, anxiolitics). The variables were selected according to the statistical significance observed in the univariate analysis (p < 0.05) or according to the association with the dependent variables observed in the literature. The Hosmer-Lemeshow test was used to assess the goodness of fit of the model.

## Results

### Population Characteristics

3588 patients were initially included in the study, of whom 399 were excluded (75 had a current diagnosis of depression, 41 had suffered pain for less than 6 weeks, 50 had a limitation when responding to the PRIME-MD questionnaire, 171 refused to participate and 199 had correctly filled in less than 80% of the information). Thus, the final number of patients studied was 3189 (response rate 88.9%) all of whom gave their written informed consent to participate in the study.

The average age of the population was 53.9 (SD: 13.3), 72.8% were female, 78.9% had completed primary or secondary education and 64% lived with a partner.

The average duration of pain was 30.8 months (median 13.9), the average intensity on the VAS was 55.9 (SD: 19.8), the most common locations of pain were the neck and back (Table 1) and the average number of pain locations was 3.5 (SD: 1.4). Also, in the 1154 patients (38.3%) where the etiology of the pain was known but the intensity was not fully explained by this cause, the most common origin of pain were musculoskeletal or connective tissue pathologies (77.8%).

**Table 1:** Characteristics of pain and its treatment in the patients studied.

Duration of pain (months)	N	1393
	Mean (SD)	30.8 (43.5)

	Median (P25; P75)	13 (6.0; 37.0)

Intensity of pain (VAS)	N	3189

	Mean (SD)	55.9 (19.8)

	Median (P25; P75)	56 (42.5; 70.0)

Pain location^a^	N = 3189	%

- Head	2063	64.7
- neck	2282	71.6
- back	2283	71.6
- limbs	1865	58.5
- joints	1954	61.3

Specific known etiology (yes^)b^	N = 1154	38.3%

- Musculoskeletal and connective tissue disorder	898	77.8
- Nervous system disorder	155	13.4
- Anxiety/Somatic disorder	117	10.1
- Metabolism disorder	70	6.1
- Vascular disorder	49	4.2
- Other disorders (neoplasias, digestive inmune, etc)	151	13.1

Current treatment^c^	N = 3004	94.2%

- Analgesics*	2729	
◦ NSAIDs	2623	96.1
◦ Opioids	290	10.6
- Psychotropics*	1262	
◦ Anxiolitics/Hypnotics	971	76.9
◦ Anticonvulsivants	99	7.8

^a ^More than 1 site was possible

^b ^More than 1 etiology was possible

^c ^More than 1 treatment was possible

SD: Standard Deviation; P25: Percentile 25; P75: Percentile 75.

* alone or in combination

Of all the patients studied, 94.2% (n = 3005) were undergoing some type of treatment for pain at the time of inclusion in the study. Of the 2729 patients under treatment with analgesics (alone or in combination with psychotropic drugs), 96.1% were taking a simple analgesic or NSAIDs, especially acetaminophen (54%) or ibuprofen (26%), and 10.6% were taking opioid analgesics (in particular tramadol, 60%) (Table 1). Of the 1262 patients taking psychotropic drugs (alone or in combination with analgesics), 76.9% were taking benzodiazepines (anxiolitics/hypnotics) (Table 1).

Out of the 3156 patients answering the first question concerning sleep, 70.3% said they slept fewer hours due to the presence of pain, and of 3141 patients answering the second question related to sleep 46.7% said that the pain symptoms were the cause of interrupted sleep.

As for the use of healthcare resources (Table 2), it should be pointed out that, due to the pain, patients had consulted a doctor at their primary care centre in the previous six weeks an average of 3.2 times; and 1546 patients (48.9% of the sample) had undergone at least 1 test to diagnose the symptoms.

**Table 2:** Use of healthcare resources in patients with unexplained pain with or without mood disorders.

Variable		Total	Without Mood Disorder	With Mood Disorder	p-value^a^
Number of visits to primary healthcare doctor due to pain in the previous 6 weeks	N	2818	549	2269	
	
	Mean (SD)	3.20 (2.16)	2.81 (2.10)	3.29 (2.17)	<0.0001
	
	Median(P25; P75)	3.0 (2.0; 4.0)	2.0(2.0; 3.0)	3.0(2.0; 4.0)	

Number of visits to a specialist due to pain in the previous 6 weeks.	N	1261	214	1047	
	
	Mean (SD)	1.14 (1.27)	0.97 (1.23)	1.18 (1.27)	0.0080
	
	Median(P25; P75)	1.0 (0.0; 1.0)	1.0 (0.0; 1.0)	1.0 (1.0; 1.0)	

Number of tests undergone for the diagnosis of pain in the previous 6 weeks.	N	1541	292	1249	
	
	Mean (SD)	1.47 (1.04)	1.50 (1.14)	1.46 (1.02)	0.6441
	
	Median(P25; P75)	1.0 (1.0; 2.0)	1.0 (1.0; 2.0)	1.0 (1.0; 2.0)	

Number of hospitalizations due to pain in the previous year	N	627	114	513	
	
	Mean (SD)	0.36 (1.05)	0.38 (1.42)	0.36 (0.95)	0.3749

	Median(P25; P75)	0.0 (0.0; 0.0)	0.0 (0.0; 0.0)	0.0 (0.0; 0.0)	

^a ^Wilcoxon Test

SD: Standard Deviation; P25: Percentile 25; P75: Percentile 75.

### Prevalence of undiagnosed mood disorder and associated factors

The prevalence of comorbid mood disorders detected by PRIME-MD was 80.4% (CI 95%: 79.0%; 81.8%). Comorbidity was higher in the female population (82.8% vs 75.4% in males; p < 0.0001) but there were no differences related to patients' age (p = 0.42).

The type of mood disorder most frequently diagnosed was major depression (56.2%), followed by minor depression (17.8%) and dysthymia (16.9%) (Table 3).

**Table 3:** Prevalence (CI 95%) of mood disorders in the population studied detected by PRIME-MD

Mood Disorder	N	Prevalence (CI 95%)
Major Depressive Disorder	1792	56.2 (54.5; 57.9)

Minor Depressive Disorder	567	17.8 (16.5; 19.1)

Dysthymia	539	16.9 (15.6; 18.2)

Bipolar Disorder	46	1.4 (1.1; 1.9)

Depression caused by medical condition, medication or drugs	80	2.5 (2.0; 3.1)

Partial remission from major depressive disorder	207	6.5 (5.7; 7.4)

The analysis of the factors associated with the presence of mood disorders in patients suffering from unexplained chronic pain can be seen in Table 4, which highlights the following factors as most strongly associated with mood disorders: being female, severe intensity of pain, longer duration of pain, pain located in the head, neck, joints or back, number of pain locations, the lack of knowledge regarding the cause of the pain, and taking anxiolytics or NSAIDs (Table 4). Being female, the presence of headaches, the cause of the pain being unknown and taking anxiolytics were risk factors associated independently with the presence of comorbid mood disorders in these patients. Similarly, the duration of pain in the previous week was a risk factor for mood disorders and taking NSAIDs was a protective factor. (Table 5).

**Table 4:** Factors associated with the presence of mood disorder (univariate analysis)

Variable	Level	N	OR	CI (95%)	p-value
Sex	Female vs Male	3076	1.51	(1.5; 1.83)	<0.0001
Age (years)	< 30	2720	1^a^		0.0920
	30-39		1.29	(0.84; 4.12)	
	40-49		1.48	(0.56; 1.93)	
	50-59		1.04	(0.73; 2.35)	
	60-69		1.31	(0.59; 1.84)	
	70-79		1.04	(0.83; 2.63)	
	≥ 80		1.86	(0.70; 2.38)	
Pain intensity	No pain (VAS ≤ 40)	3189	1^a^		<0.0001
	Moderate pain (VAS 41-70)		1.37	(1.11; 1.68)	
	Severe pain (VAS >70)		1.95	(1.51; 2.53)	
Duration of pain (months)	> 2 years	1393	1^a^		0.2824
	< 6 months vs >2 years		1.21	(0.85; 1.73)	
	Between 6-12 months vs > 2 years		1.28	(0.88; 1.87)	
	Between 1-2 years vs > 2 years		0.91	(0.63; 1.31)	
NSAIDs	Yes vs No	3189	0.67	(0.52; 0.86)	0.0018
Analgesic opioids	Yes vs No	3189	1.07	(0.79; 1.46)	0.6600
Anticonvulsionants	Yes vs No	3189	1.03	(0.62; 1.71)	0.9181
Anxiolitics	Yes vs No	3189	1.56	(1.27; 1.91)	<0.0001
Headache	Yes vs No	3189	1.66	(1.39; 1.99)	<0.0001
Neck pain	Yes vs No	3189	1.70	(1.41; 2.04)	<0.0001
Back pain	Yes vs No	3189	1.66	(1.38; 1.99)	<0.0001
Limb pain	Yes vs No	3189	1.24	(1.04; 1.48)	0.0165
Joint pain	Yes vs No	3189	1.52	(1.27; 1.81)	<0.0001
Identification of the cause of pain	No vs Yes	3011	1.72	(1.44; 2.06)	<0.0001
Pain duration in the last week (percentage of the day in pain)		3189	1.02	(1.01; 1.02)	<0.0001

Number of pain sites	1	2959	1^a^		<0.0001
	2		1.21	(0.87;1.67)	
	3		1.71	(1.23; 2.36)	
	4		1.91	(1.36;2.68)	
	5		2.54	(1.86;3.47)	

^a ^reference category

**Table 5:** Logistic regression model of the variables associated with the presence of mood disorder in patients with pain (OR adjusted ± CI 95%) N = 2907

Variable	Level	OR	CI (95%)	p-value
Sex	Male vs Female	1.48	(1.22; 1.81)	0.0001
NSAIDs	Yes vs No	0.67	(0.51; 0.88)	0.0051
Anxiolitics	Yes vs No	1.07	(1.11; 1.66)	0.0086
Headache	Yes vs No	1.39	(1.15; 1.70)	0.0008
Duration of pain in the last week		1.01	(1.01;1.02)	0.0001
Identification of the cause of pain	No vs Yes	1.74	(1.45; 2.10)	<0.0001

Hosmer-Lemeshow = 11.21 p = 0.189 χ^2 ^= 160.46; p < 0.0001

### Use of healthcare resources in patients with chronic pain and mood disorders

As displayed in Table 2, the use of healthcare resources was significantly higher in patients with comorbid unexplained pain and any mood disorder compared to those only suffering from unexplained pain. Comorbid patients consulted both GPs and specialists with greater frequency. However, no differences were observed between the groups regarding the number of diagnostic tests carried out or hospitalization rates (Table 2)

## Discussion

This study was undertaken to investigate the comorbidity of unrecognized mood disorders and unexplained chronic pain in primary care patients, as well as to determine the possible factors influencing such a relationship. In addition, the study also investigated the use of healthcare resources as a consequence of this comorbidity.

The most important findings related to the aim of our study were: a) the existence of a high prevalence of undiagnosed mood disorders in patients suffering from unexplained chronic pain complaints evaluated in a primary care setting; b) the frequent presence in these patients of other mood disorders, different to MDD c) a greater susceptibility of women to mood disorders; d) a direct relationship between the prevalence of mood disorders and the duration of pain in the previous week; e) an absence of association between the intensity and number of pain-sites; f) a higher comorbidity if the reasons for suffering pain are unknown and, g) the observation of an increased use of healthcare services in patients with such comorbidity.

Our results confirm previous studies reporting that the presence of painful physical symptoms increases the likelihood of a diagnosis of a mood or anxiety disorder by as much as 3-fold [24]. Also, they are in agreement with data reported by Tang et al [25] who concluded that in chronic back pain patients, experimentally induced negative mood increased self-reported pain and decreased tolerance, with positive mood having the opposite affect.

Despite being very common in primary care settings, mood disorders are frequently undetected. A number of studies have found that about half of the patients who experience major depression are not diagnosed by their primary care physicians [26]. In addition, 50% to 80% of patients with depression initially have a painful physical symptom [11]. These data would suggest that these individuals are considerably less likely to receive an accurate psychiatric diagnosis, will have a limited access to specific treatments and a poor outcome. Rijswijk and colleges [27] report some factors which serve as barriers for suitable mental health care for GPs, and they mention the difficulties in distinguishing between psychological problems and a complete psychiatric disorder, or the difficulties in assessing severity of the disorder, as the most important. In this regard, it is worth highlighting the high percentage of patients in this study who had not received a diagnosis of mood disorder.

One result found in this study, and which, to our knowledge has not been reported by other authors is the high frequency of minor depression or dysthymia observed in the patients. The presence of these mood disorders in almost 35% of the patients could be explained by the use of the PRIME-MD, which is better able to detect these processes in primary healthcare than instruments used in other studies.

Our study also found that women suffer a greater risk of experiencing a comorbid pain-mood disorder. This result is congruent with findings by Ohayon and Schatzberg [6]. Campbell et al [3] and Keogh et al [28] also suggested that there is evidence that comorbid chronic pain and depression is more likely to occur in women than in men. However, Arnow et al [29] found that the degree of the comorbidity between chronic pain and depression does not differ according to age or gender. Although differences in pain reporting between women and men are partly attributable to social conditioning and to psychosocial factors, some experimental studies have described sex differences in sensitivity to noxious stimuli, suggesting that biological mechanisms underlie such differences [30].

In this study, a direct association was found between the number of pain sites and the presence of mood disorder, as measured by the crude OR, but this relationship disappeared after adjustment by other variables. These data are contradictory when we refer to previous studies [2]. Gureje et al. [31], who analyzed data from the World Health Organization to examine persistent pain in primary care patients around the world, found that half of patients who experienced persistent pain at onset continued to have persistent pain 12 months later. In this study, the best independent predictor of persistent pain was the number of pain sites. Moreover, in a more recent study, Gureje et al. [32] found increased rates of depression and anxiety in patients complaining of multi-site pain. The cause of such contradictions may result from methodological differences, related to differences in design, populations studied and also probably due to the fact that we have adjusted for intensity and duration of pain.

We found a higher rate of comorbid pain-mood disorder if the reasons for suffering pain are unknown, and also when a precise diagnosis of the cause of pain is lacking. In some studies, a higher occurrence of depression is reported if the etiology of the pain condition is medically unexplained when compared to patients with a more defined pain disorder, such as neuropathy [33]. Marazziti et al [33] explain that patients with unexplained pain have a higher likelihood of reporting catastrophic thoughts and they tend to think that the origin of their pain is a mystery; they feel that they have lost control and that their physician does not believe their pain to be real.

Finally, we found an increased use of health care services when pain is comorbid with mood disorder. This result is replicated very often in other studies [34]. The possible explanation of this coincidence is that the presence of mood disorder complicates the management of patients with pain, resulting in a poor outcome, i.e. longer duration of pain and a greater likelihood of non recovery [4]. In this regard, some GPs declare factors such as depression or anxiety to be among the most significant and problematic complications in treating patients with chronic pain [35].

It is necessary to know the complex biological relationships between chronic pain and depression to better understand the frequency of this comorbidity. The convergence of depression and pain is reflected in the circuitry of the nervous system. Brain pathways that handle the reception of pain signals, including the limbic system, use some of the same neurotransmitters involved in the regulation of mood, especially serotonin and norepinephrine. When an alteration occurs in these pathways pain is intensified, along with depression and anxiety. This is one of the reasons why, in many cases, antidepressants - mainly those that inhibit the reuptake of norepinephrine or serotonin - are used, either alone or in combination with analgesics, to treat chronic pain [15].

A limitation of our study is that the occurrence of other chronic medical or psychiatric conditions, has not been assessed and they are sometimes related to depression and often prevalent among people with chronic pain [36,37]. Furthermore, the survey conducted was a cross-sectional study and consequently does not provide information about the direction of causality between unexplained chronic pain and mood disorders. We can therefore only postulate on the possible way in which the association between pain and mood disorder is established. Some explanations have been offered; it has even been suggested, that certain pain conditions, such as migraine [38], share a common predisposition with some psychiatric disorders [32]. However, more follow-up studies are necessary to improve our understanding of chronic pain-mood disorder relationships in primary care.

We have to point out the possible bias related to the subjective assessment of pain by the GP in order to consider the patients as being affected by unexplained chronic pain and thus to select the patients to be included in the study. However, this bias should be small because of the training given to the GP, and because of the use of objective assessment of pain by a VAS.

Another limitation could be the lack of a confirmation of the diagnosis by means of a structured psychiatric interview. However the PRIME-MD is an instrument with tried and tested psychometric properties.

A strength of this study is that our results are based on large datasets collected from a broad range of primary care centres by means of objective assessment of pain and using international criteria for mood disorder diagnosis from a validated tool.

## Conclusion

There is a very high under diagnosed prevalence of different mood disorders in patients with unexplained chronic pain visiting a primary care physician, leading to dissatisfaction with treatment processes and outcomes. Consequently, it seems necessary to explore this condition more regularly in general practice in order to achieve accurate diagnoses and to select the appropriate treatment.

## Competing interests

The authors declare that they have no competing interests.

## Authors' contributions

LA has made substantial contributions to the conception and design of the paper, and has given his final approval of the version to be published. Likewise, he had full access to all of the data in the study and takes responsibility for the integrity of the data and the accuracy of the data analysis. IF has made substantial contributions to the conception and design of the paper and the analysis and interpretation of data. She has also been involved in drafting the manuscript. JC has made substantial contributions to the coordination of the study and helped to write the manuscript. PD-F has made substantial contributions to the coordination of the study. JAM has been involved in drafting the manuscript and reviewing it critically for important intellectual content.

All the authors have read and approved the final manuscript.

## Pre-publication history

The pre-publication history for this paper can be accessed here:

http://www.biomedcentral.com/1471-2296/11/17/prepub
